# Reprogramming the immune orchestra for glioblastoma: oncolytic viruses and the B-cell connection

**DOI:** 10.1038/s41423-026-01401-2

**Published:** 2026-03-19

**Authors:** Costanza Dieli, Marta Di Simone, Francesco Dieli, Serena Meraviglia

**Affiliations:** 1 Central Laboratory of Advanced Diagnosis and Biomedical Research (CLADIBIOR), University of Palermo, Palermo, Italy; 2 Department of Precision Medicine in Medical, Surgical and Critical Care (MePreCC), University of Palermo, Palermo, Italy; 3 Department of Health Promotion, Mother and Child Care, Internal Medicine and Medical Specialties (ProMISE), University of Palermo, Palermo, Italy; 4 Department of Biomedicine, Neurosciences and Advanced Diagnosis, University of Palermo, Palermo, Italy

**Keywords:** Oncology, Immunology

Glioblastoma multiforme (GBM) is a lethal primary brain cancer for which treatment has limited options, characterized by systemic and local immunosuppression that contributes to the malignancy's aggressiveness and resistance to therapy. In a recent study published in *Cellular and Molecular Immunology* [1], Han et al. reported that intravenously administered oncolytic alphavirus M1 not only suppressed GBM growth but also reversed GBM-induced systemic immunosuppression.

Glioblastoma (GBM) remains the most aggressive primary brain tumor in adults and continues to pose one of the most formidable challenges for cancer immunotherapy [2]. Despite major advances in understanding tumor–immune interactions and the clinical success of immune checkpoint blockade (ICB) across multiple solid malignancies, GBM has consistently failed to derive durable benefit from immunotherapeutic strategies [3]. This resistance cannot be fully explained by the relatively low mutational burden of gliomas. Instead, GBM is characterized by a profound and multilayered immunosuppressive program that extends beyond the tumor microenvironment (TME) and affects systemic immune compartments [4]. Peripheral lymphopenia, splenic atrophy, impaired antigen presentation, dysfunctional T-cell priming, and widespread T-cell exhaustion are well-documented features of GBM patients and represent central barriers to effective immunotherapy [4].

Over the past decade, numerous strategies have been explored to overcome immune resistance in GBM, including vaccination approaches, adoptive T-cell therapies, modulation of myeloid populations, and combinatorial checkpoint inhibition. While many of these approaches have demonstrated immunological activity in preclinical settings, clinical outcomes have remained modest and heterogeneous [3].

Oncolytic virotherapy has emerged as a promising immunotherapeutic strategy for GBM because of its ability to reshape the GBM microenvironment, primarily through three mechanisms [5]. First, oncolytic viruses (OVs) replicate selectively within neoplastic cells, leading to immunogenic cell death that activates innate immunity through pathogen-associated molecular pattern (PAMP) receptors [6]. Second, oncolysis-facilitated release of tumor-associated antigens (TAAs) leads to the recruitment of APCs to the TME and the induction of adaptive antitumor responses [5, 6]. Moreover, OVs induce the secretion of proinflammatory cytokines, which increase MHC expression on both infected and bystander tumor cells, making gliomas more recognizable to the immune system [5]. In phase I-II clinical trials, locoregional delivery of OVs has demonstrated favorable tolerability and the ability to modulate the tumor microenvironment and trigger antitumor immune responses against GBMs [5]. However, durable antitumor responses remain rare, suggesting that local immune activation alone may be insufficient when systemic immune competence and effective T-cell priming are compromised.

A common conceptual limitation of many immunotherapeutic approaches in GBM is the assumption that productive antitumor immunity must be initiated within the tumor or its draining lymph nodes. In reality, the GBM TME is profoundly hostile to immune activation. Antigen-presenting cells are functionally impaired, metabolic constraints limit effector function, immunosuppressive cytokines such as IL-10 and TGF-β are abundantly produced, and chronic antigen exposure drives progressive T-cell exhaustion. Under these conditions, increased antigen availability does not necessarily translate into effective cytotoxic T-cell responses. These observations have prompted growing interest in alternative anatomical sites and immune circuits that support antigen presentation and T-cell activation outside the suppressive tumor niche.

In this context, a recent study by Han and colleagues revealed a conceptually important shift in how antitumor immunity in GBM can be reactivated [1]. Using an intravenously administered oncolytic alphavirus M1, the authors demonstrated that systemic virotherapy not only suppressed glioma growth but also reversed key features of GBM-induced systemic immunosuppression. Rather than focusing exclusively on intratumoral immune remodeling, this study identifies the spleen as an indispensable site for antitumor immune priming and reveals a previously underappreciated role for splenic B cells in driving CD8⁺ T-cell responses.

**Fig. 1:**
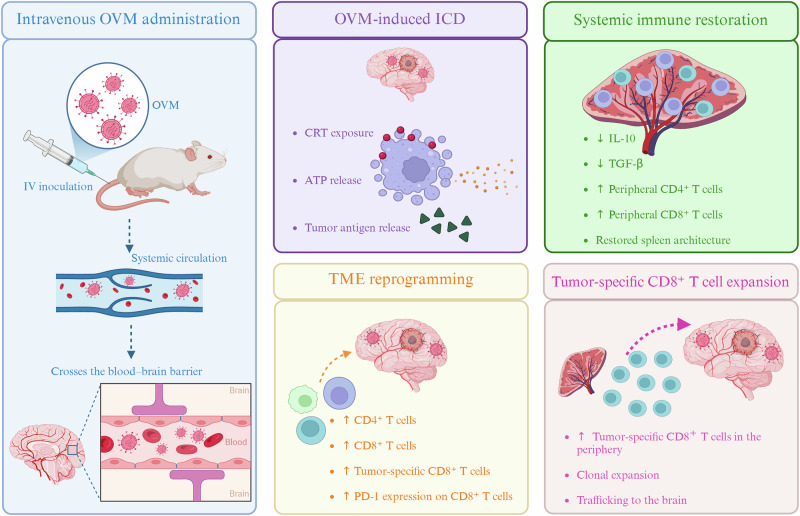
Systemic immunomodulatory effects of intravenous OVM in GBM. Intravenous administration of oncolytic virus M1 (OVM) enables blood–brain barrier penetration and induces immunogenic cell death (ICD), characterized by calreticulin (CRT) exposure, ATP release, and tumor antigen release. This promotes tumor microenvironment (TME) reprogramming and restores systemic immune competence, with reduced interleukin-10 (IL-10) and transforming growth factor-β (TGF-β) levels. OVM treatment drives the clonal expansion of tumor antigen–specific CD8⁺ T cells and their trafficking to the brain in glioblastoma (GBM)

One of the most compelling findings of this work is that splenectomy completely abolishes the therapeutic efficacy of M1 macrophages. In glioma-bearing mice, GBM induces marked splenic atrophy, disruption of lymphoid architecture and depletion of circulating T cells, which is consistent with systemic immune collapse [4]. The intravenous administration of M1 restored the splenic size, cellularity, and immune composition (Fig. 1). The complete loss of therapeutic benefit following splenectomy establishes a causal relationship between splenic immune function and tumor control, underscoring that the spleen is not merely a passive reservoir for immune cells or a filter for circulating viruses but also a central hub orchestrating systemic antitumor immunity.

The role of the spleen in cancer immunology has long been ambiguous. While it can serve as a reservoir for immunosuppressive myeloid populations that support tumor progression, accumulating evidence indicates that it also plays a crucial role in sustaining antitumor T-cell pools and mediating responses to immunotherapy [7, 8]. Intermediate states of exhausted CD8⁺ T cells residing in the splenic white pulp have been shown to dictate responsiveness to ICB, highlighting the spleen as a site of immune plasticity. The present study extends this paradigm by demonstrating that the spleen also functions as a primary site of antigen presentation and T-cell priming during oncolytic virotherapy [1].

A particularly unexpected and original aspect of this work is the identification of splenic, marginal zone-derived B cells as critical mediators of CD8⁺ T-cell priming [1]. While dendritic cells are traditionally regarded as the principal antigen-presenting cells responsible for cross-presentation, this study provides multiple lines of evidence that B cells assume this role following M1 treatment. Single-cell transcriptomic analyses revealed enhanced interactions between B cells and T cells in the spleen after virotherapy, accompanied by the upregulation of antigen processing and presentation pathways, costimulatory molecules, and immune synapse components [1]

The results of functional experiments further substantiate the direct role of B cells in CD8⁺ T-cell activation. B cells isolated from virus-treated mice efficiently activated CD8⁺ T cells in a contact-dependent, MHC class I-restricted manner, which is consistent with bona fide cross-presentation of tumor antigens. Importantly, depletion of B cells or genetic ablation of B-cell compartments completely abrogates M1-mediated antitumor efficacy, despite preserved viral oncolysis. These findings firmly establish B cells as essential contributors to the therapeutic response and prevent immune-mediated tumor control from direct viral cytotoxicity [1].

**Fig. 2:**
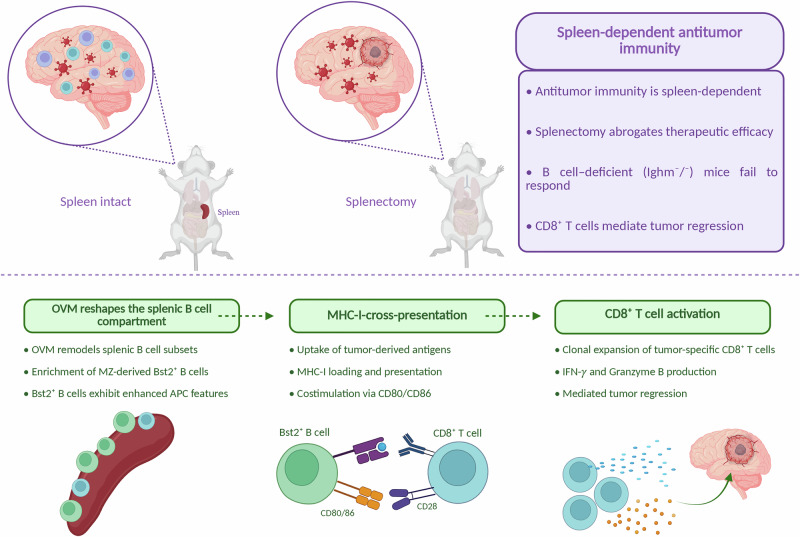
Bst2⁺ B cells mediate splenic priming of CD8⁺ T cells. OVM efficacy in glioblastoma (GBM) requires an intact spleen, as splenectomy or B-cell deficiency abrogates tumor control, and CD8⁺ T cells mediate tumor regression. OVM reshapes the splenic B-cell compartment, promoting enrichment of marginal zone (MZ)-derived Bst2⁺ B cells with enhanced antigen-presenting capacity. These Bst2⁺ B cells cross-present tumor-derived antigens via MHC-I and provide costimulation through CD80/CD86, leading to the activation and clonal expansion of tumor-specific CD8⁺ T cells. Activated CD8⁺ T cells acquire effector functions, including interferon-γ (IFN-γ) and granzyme B (GzmB) production, ultimately supporting antitumor immunity

The authors further refined this mechanism by identifying a distinct subset of B cells expressing bone marrow stromal cell antigen 2 (Bst2) with superior cross-presentation capacity. These Bst2⁺ B cells originate from marginal zone B cells, a population strategically positioned to capture blood-borne antigens. Upon M1 exposure, marginal zone B cells differentiate into Bst2⁺ cells—a process most likely mediated by type-I interferon—which is characterized by enhanced proteasome-dependent antigen processing, upregulation of the expression of MHC class I and costimulatory molecules, and a robust ability to activate tumor-specific CD8⁺ T cells (Fig. 2). Adoptive transfer experiments demonstrated that the reconstitution of Bst2⁺ B cells is sufficient to restore antitumor immunity and prolong survival in B-cell-deficient glioma-bearing mice, underscoring the functional relevance of this subset.

Together, these findings delineate a coherent sequence of events in which intravenous oncolytic virotherapy induces immunogenic tumor cell death, promotes the release and systemic dissemination of tumor antigens, licenses splenic B cells for cross-presentation, and drives the expansion and activation of tumor-reactive CD8⁺ T cells [1, 8]. These primed T cells subsequently travel to the brain and mediate tumor control. This spleen-to-brain immunological axis effectively bypasses the immunosuppressive constraints of the TME and challenges the long-standing assumption that antitumor T-cell priming must occur locally within the tumor or its draining lymphatics.

This framework also provides mechanistic insight into the observed synergy between M1 and PD-1 blockade [1, 9]. In GBM, checkpoint inhibition alone is largely ineffective, in part because of the scarcity of functional tumor-reactive T cells. M1 treatment expands and activates CD8⁺ T cells systemically, leading to increased PD-1 expression because of activation rather than terminal exhaustion. Under these conditions, PD-1 blockade becomes functionally relevant, unleashing cytotoxic activity within a newly generated pool of tumor-reactive T cells [1]. Thus, oncolytic virotherapy acts not simply as a cytolytic agent but also as an immune sensitizer that restores the prerequisites for effective checkpoint inhibition.

This study also clarified the relative contribution of direct viral oncolysis versus immune-mediated tumor control. Although M1 macrophages can cross the blood–brain barrier and selectively infect glioma cells, depletion of CD8⁺ T cells completely abrogates therapeutic efficacy, indicating that adaptive immunity is indispensable for durable tumor control. This distinction is critical for understanding how oncolytic virotherapy should be integrated into combination treatment strategies.

Beyond its mechanistic insights, this work adds important nuance to the ongoing debate regarding the role of B cells in GBM. While intratumoral B cells are often associated with immunosuppressive functions, including regulatory B-cell activity and plasma cell differentiation, the present study highlights a spatially and functionally distinct B-cell compartment that operates outside the tumor and exerts potent immunostimulatory activity [1]. These findings reinforce the notion that the impact of B cells in cancer is highly context-dependent and shaped by anatomical location and inflammatory cues.

From a translational perspective, several implications emerge. First, the data provide a strong rationale for the systemic, rather than intratumoral, delivery of oncolytic viruses in GBM. Second, they identify splenic B cells—and specifically cross-presenting subsets—as potential targets or tools for immunotherapeutic intervention, including the development of B-cell-based antigen presentation strategies. Third, these findings suggest that successful immunotherapy in GBM may require deliberate restoration of systemic immune competence before or in conjunction with checkpoint blockade.

Moreover, important limitations remain. The reliance on murine models raises questions regarding translatability, particularly with respect to the existence and functional equivalence of Bst2⁺ B cells in humans. Moreover, the upstream molecular signals that drive the differentiation of marginal zone B cells into potent cross-presenting cells remain incompletely defined. Finally, although early clinical data support the safety of systemic oncolytic virotherapy, careful evaluation of off-target effects and patient selection will be essential as these approaches move toward clinical application in GBM.

In summary, the study by Han and colleagues strongly supports an emerging shift in how antitumor immunity is conceptualized in GBM, emphasizing the importance of systemic immune reprogramming and extratumoral antigen presentation. By revealing a spleen-centered, B-cell-dependent mechanism of CD8⁺ T-cell priming, this work challenges strictly tumor-centric views of antitumor immunity and provides a conceptual framework for integrating oncolytic virotherapy with immune checkpoint blockade.
